# Evaluation of mucosal adjuvants to chitosan-nanoparticle-based oral subunit vaccine for controlling salmonellosis in broilers

**DOI:** 10.3389/fimmu.2025.1509990

**Published:** 2025-02-03

**Authors:** Raksha Suresh, Shekoni Olaitan Comfort, Sara Dolatyabi, Jennifer Schrock, Mithilesh Singh, Gourapura J. Renukaradhya

**Affiliations:** Center for Food Animal Health, Department of Animal Sciences, College of Food, Agricultural, and Environmental Sciences, The Ohio State University, Wooster, OH, United States

**Keywords:** *Salmonella* Enteritidis, broiler chickens, adjuvants, oral vaccine, mucosal immunity

## Abstract

Salmonellosis, a gastrointestinal disease, continues to be one of the major public health concerns worldwide. Poultry meat and eggs are recognized as the major source of *Salmonella* food poisoning in humans. Our study evaluated the protective efficacy of mannose-conjugated chitosan-nanoparticle (mChitosan-NP)-based subunit vaccine, consisting of immunogenic outer membrane proteins and flagella of *Salmonella* Enteritidis [mChitosan (OMP+FLA)/FLA-NP], coadministered orally with potent mucosal adjuvants to reduce the colonization of *S.* Enteritidis in the intestines of broiler chickens. We evaluated the adjuvant effects of three different doses of two well-known mucosal adjuvants, c-di-GMP (stimulator of interferon gene agonist) and whole cell lysate (WCL) of *Mycobacterium smegmatis*, to improve the efficacy of mChitosan (OMP+FLA)/FLA-NP vaccine. Our data reaffirmed the potent adjuvanticity of both of these adjuvants and identified their optimal dose when entrapped in mChitosan-NP to potentiate the immunogenicity and efficacy of orally delivered mChitosan (OMP+FLA)/FLA-NP vaccine. The physical characteristics of mChitosan (OMP+FLA)/FLA-NP, mChitosan-GMP/FLA-NP, and mChitosan-WCL/FLA-NP formulations revealed a high positive charge (Zeta potential +20–25 mV), size 235–260 nm, and polydispersity index 0.35–0.52, which are conducive for oral delivery. The efficacy in chickens that received oral administration with a combination of the vaccine-adjuvant formulations was evaluated by challenging with *Salmonella* Enteritidis. Our data showed that mChitosan (OMP+FLA)/FLA-NP WCL at 10 µg/dose formulation consistently reduced the *S.* Enteritidis load by over 0.5 log_10_ comparable to a commercial live vaccine at post-challenge days 4 and 10. Immunologically, we observed enhanced systemic and mucosal antibody and cellular (B cells and T-helper cells) immune responses as well as upregulation of expression of immune cytokine genes IFN-γ, TGF-β, and IL-17 in the cecal tonsils of adjuvanted mChitosan-NP *Salmonella*-subunit-vaccinated birds. Overall, particularly the mucosal adjuvant WCL consistently enhanced the efficacy of mChitosan (OMP+FLA)/FLA-NP vaccine by inducing effective immune responses.

## Introduction

Salmonellosis, a public health concern, is a disease caused by a bacterium of the genus *Salmonella*. This is a gram-negative, rod-shaped, facultatively anaerobic bacteria. The latest report by FoodNet highlights *Salmonella* and *Campylobacter* as the leading causes of foodborne enteric infections (1). *Salmonella enterica* species is subdivided into six subspecies (2), with subspecies I causing most human infections, particularly the serotypes Enteritidis, Typhimurium, Typhi, and Choleraesuis. *Salmonella* is often transmitted to the human food chain through poultry meat and eggs.

The biosecurity measures followed in poultry farms to control salmonellosis include using high-quality feed and maintaining hygiene, but they were often found to be expensive and insufficient. Vaccination is a viable strategy to control salmonellosis. However, an ideal vaccine should be cost-effective and broad-spectrum, with the ability to elicit long-lasting immunity. Live *Salmonella* vaccines are commonly used and administered through drinking water or spray methods, but they pose an environmental risk due to their entry into the human food chain (3). To mitigate this, the vaccine should not be administered at least 21 days before the slaughter of poultry. This extended interval between the last vaccination and slaughter can potentially increase the risk of salmonellosis. While killed *Salmonella* vaccines are safe, they are not as effective as the live vaccines in controlling *Salmonella* outbreaks despite repeated administration in chickens by intramuscular injection (4). Furthermore, injectable killed vaccines are labor-intensive and may also impact meat quality, which is a major concern in broilers. Therefore, current vaccines and vaccination strategies are inefficient, warranting the need for the development of innovative vaccine platforms and delivery strategies. Subunit vaccines using conserved antigens like outer membrane proteins (OMP) and flagellin (FLA) of *Salmonella* showed a promise by eliciting robust immune responses against salmonellosis (5, 6). In our previous studies, *Salmonella* subunit oral vaccine using the chitosan-nanoparticle delivery platform [chitosan (OMP+FLA)/FLA-NP] was found to be effective in reducing the colonization of *Salmonella* in the intestines of broilers by eliciting innate and specific adaptive immune responses (7–11). This oral vaccine delivery platform has distinct features, such as the fact that it makes use of chitosan, is non-toxic, is biocompatible, and is a well-known mucoadhesive agent with inherent adjuvant properties. In addition, mannose conjugation of chitosan helps in targeting the vaccine to dendritic cells, leading to the induction of enhanced adaptive immunity (7).

In this study, to further improve the efficacy of mChitosan (OMP+FLA)/FLA-NP vaccine, we coadministered the vaccine with potent mucosal adjuvants such as whole cell lysate (WCL) of *Mycobacterium smegmatis* and cyclic di-GMP (c-di-GMP). The adjuvant WCL stimulates B cells and macrophages, promoting antibody production (12), and c-di-GMP is an immunomodulator that promotes antigen uptake and cytokine production, leading to improved adaptive immune responses (13).

Furthermore, the antigen dose in a vaccine formulation is crucial for its efficacy dynamics because a high antigen dose is detrimental to vaccine-induced protection (14, 15). An earlier study elucidated that a high vaccine dose is detrimental because it leads to the production of more specialized CD4 T cells which are less effective in reaching the target site and responding to the antigen (15). Additionally, another study underscored that the use of higher amounts of antigen in vaccine could induce immunotolerance, thereby suppressing immune responses (3, 14). Even in our previous study, a higher dose (50 *versus* 10 µg/dose) of OMP and FLA antigens delivered orally in chitosan-NPs did not elicit dose-dependent enhanced immune response and efficacy in reducing *Salmonella* load in the intestines of broilers (10). Thus, it is critical to choose an optimum dose of the antigen in a vaccine. In this study, our objective was to determine the potent adjuvanticity and optimum dose of the mucosal adjuvant for potentiating the efficacy of mChitosan (OMP+FLA)/FLA-NP oral vaccine against the colonization of *Salmonella* in broilers. Therefore, we used a fixed single dose of 20 µg of pooled OMP+FLA antigens entrapped in mChitosan-NP, coadministered with three different doses of two mucosal adjuvants WCL and c-di-GMP. These vaccination strategies may lead to the development of an effective vaccine formulation to mitigate salmonellosis in poultry, thus addressing the ongoing challenges posed by *Salmonella* food poisoning.

## Materials and methods

### Extraction of OMP

*Salmonella* Enteritidis (SE) was cultured in tryptic soy broth until the culture reached an optical density (OD) of 1.0. The bacterial pellet was collected by centrifugation, washed with Tris-HCl, and kept at -80°C overnight. After thawing, the bacterial pellet was heated at 75°C for 10 min to kill the bacteria. The heat-killed bacteria were sonicated and centrifuged at 8,000 *g* for 10 min. The supernatant was ultracentrifuged at 108,726 *g* for 30 min, and the resulting pellet was washed in Tris-HCl containing 2% Triton X-100. The pellet was incubated for 30 min at room temperature, followed by ultracentrifugation at 100,000 *g* for 2 h. The final pellet was dissolved in phosphate-buffered saline (PBS), and the detailed procedure followed was as described previously (16, 17).

### Extraction of flagella

*Salmonella* Enteritidis was grown in brain heart infusion broth at 37°C without shaking. The bacterial pellet was collected and washed once in PBS and treated with 3 M potassium thiocyanate for 2 h at 4°C with magnetic stirring. The preparation was ultracentrifuged at 35,000 *g* for 30 min. The supernatant containing flagellin protein was dialyzed against PBS for 12 h, followed by another 12 h in Milli-Q water (17).

### Preparation of mannose-chitosan-based adjuvant and *Salmonella* subunit antigens

(i) Preparation of c-di-GMP-NP and WCL-NP adjuvant formulations: The mannose-modified chitosan-NP entrapped with either the whole cell lysate of *Mycobacterium smegmatis* (WCL) or c-di-GMP (InvivoGen) was prepared using the ionic gelation method as previously described with few modifications (7, 18, 19). Briefly, 38 mg of lyophilized mannose-conjugated chitosan was dissolved in Milli-Q water at a concentration of 1 mg/mL, and the pH was adjusted to 4.3. To this solution, 3.8 mg of WCL or c-di-GMP (dissolved in 3.8 mL of 10 mM MOPS buffer at pH 7.4) was added dropwise using an insulin syringe, and the mixture was kept under magnetic stirring. Subsequently, 9.8 mg of sodium tripolyphosphate (TPP) dissolved in 19 mL of Milli-Q water was added dropwise using an insulin syringe. After 10 minutes of stirring, 950 µg of flagellin protein in 1 mL of MOPS buffer was added to the formulation. The resulting NPs were extracted as a pellet by centrifugation at 10,976 *g* for 30 min. The WCL protein entrapment efficiency was calculated by estimating the amount of protein present in the supernatant, obtained in the final high-speed centrifugation step by using the bicinchoninic acid (BCA) method. For c-di-GMP entrapped NPs, the entrapment efficiency was performed by spectrophotometry recording of the supernatant at OD 260/280 nm. The two adjuvant formulations were lyophilized and reconstituted in Milli-Q water before use in the vaccination of chickens.

(ii) Preparation of mannose chitosan (OMP+FLA)/FLA vaccine formulation: The mChitosan-NP entrapped OMP+FLA formulation was prepared by following the same procedure used for adjuvant mChitosan-NP formulations by the ionic gelation method. Briefly, 72 mg of lyophilized mannose-conjugated chitosan was dissolved in 72 mL of Milli-Q water under magnetic stirring, and the pH was adjusted to 4.3. To this solution, 7.2 mg of pooled OMP and FLA (3.6 mg OMP + 3.6 mg FLA) dissolved in 7.2 mL of 10 mM MOPS (pH 7.4) was added. Subsequently, 6 mg of TPP dissolved in 36 mL of Milli-Q water was added. Finally, 1.8 mg of FLA dissolved in 1 mL of 10 mM MOPS buffer was added dropwise. The resulting mChitosan (OMP+FLA)/FLA-NP was extracted as a pellet through centrifugation at 10,976 *g* for 30 min. The entrapment efficiency of vaccine proteins in the formulation was calculated by estimating the amount of protein in the supernatant by using the BCA method.

The physical nature of mChitosan-NP entrapped with OMP and FLA or both of the adjuvants was determined using the Malvern Zetasizer as described previously (7, 19).

### Immunizations and challenge studies in chicken

**Figure 1:**
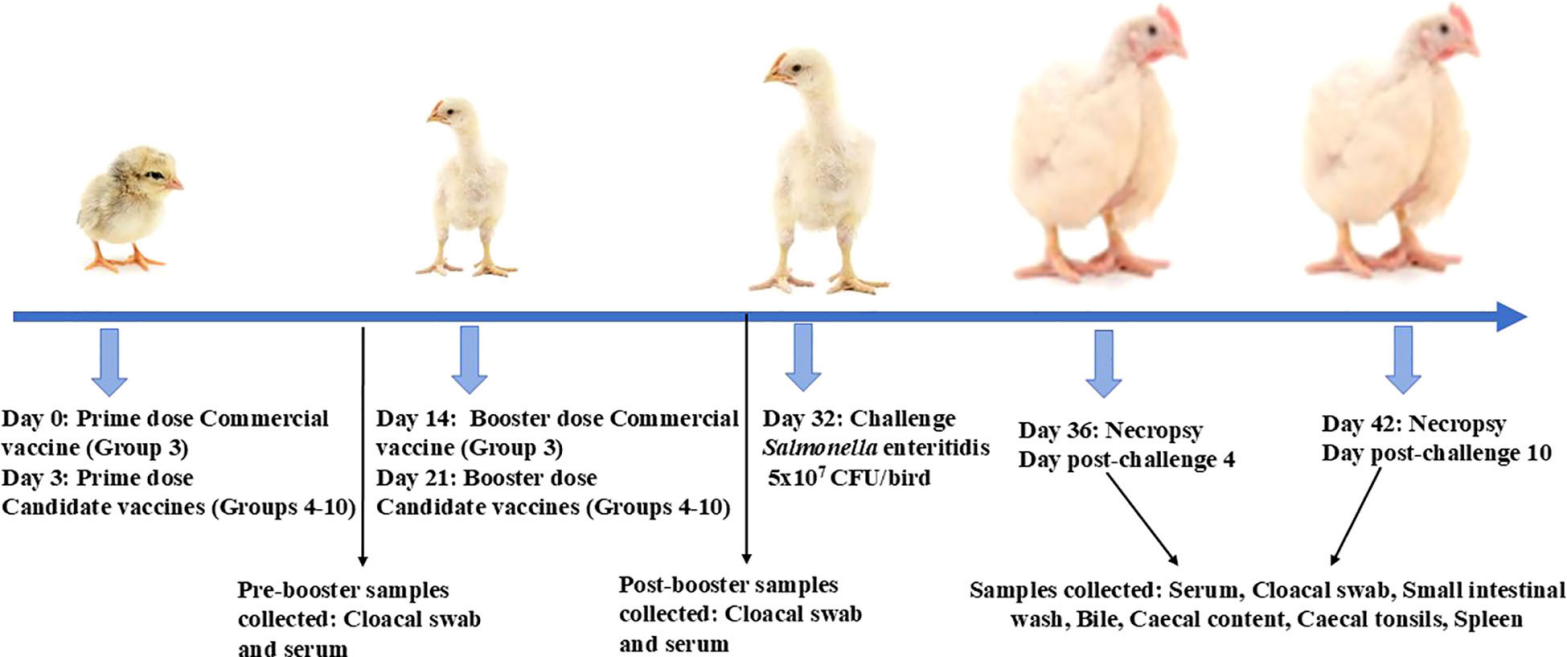
Experimental design. Broiler birds were prime-boost-vaccinated with WCL or GMP-adjuvanted mChitosan (OMP+FLA)/FLA vaccine orally, beginning at age 3 days, boosted 3 weeks later, and challenged with *S*. Enteritidis at day 32; necropsy was performed at DPC4 and DPC10. The samples were collected at pre-booster, at post-booster, and at DPC4 and DPC10 for immune and bacterial analysis.

Cornish cross broiler chicks were received on the day of hatching from an Ohio commercial hatchery. The birds were randomly screened for the presence of *Salmonella* upon arrival and grouped randomly into 10 groups (10 to 13 birds per group). The prime vaccination was done on day 3, and the booster dose was given on day 21 through oral gavage as described previously (10). Cloacal swabs (for estimation of secretory IgA) and serum samples (for IgY estimation) were collected both before the booster (pre-booster) and after the booster at 1 day before the challenge infection (post-booster). Each vaccine dose contained 10 µg each of OMP and FLA proteins (total 20 µg/dose) encapsulated and surface-coated with FLA on mChitosan-NP. This vaccine was coadministered with one of the three doses of the adjuvants WCL or c-di-GMP encapsulated in mChitosan-NP at 2.5, 10, and 50 µg per dose. The commercial vaccine Poulvac ST, a live *Salmonella* vaccine from Zoetis, was administered to a group of control birds at the age of 1 day and 2 weeks as per the manufacturer’s instructions. At the age of 5 weeks, all of the birds, except the mock control group, were challenged orally with a pre-titrated dose of SE 5 ×10^7^ colony-forming units (CFU)/bird. The necropsy (*n* = 5 to 7 birds per group) was conducted on days 4 and 10 post-challenge (DPC4 and DPC10). During necropsy, samples of bile, blood, cloacal swab, cecal tonsils, small intestinal wash, and cecal contents were collected and stored at −80°C until analyzed. Splenocytes were isolated from the spleen and used in the phenotypic analysis of immune cells by flow cytometry (**Figure 1** and **Table 1**).

**Table 1:** Salmonella vaccination regimen and challenge study design in broilers.

Group	Vaccine formulations	First dose	Second dose	Challenge age	Number of birds DPC4	Number of birds DPC10
1	Mock	NA	NA	NA	6	6
2	Mock-Challenge	NA	NA	5^th^ week	6	5
3	Commercial vaccine + Challenge	Day 0	Day 14	5^th^ week	6	6
4	mChitosan (OMP+FLA)/FLA-NP + Challenge	Day 3	Day 21	5^th^ week	6	6
5	mChitosan (OMP+FLA)/FLA -NP WCL 2.5 + Challenge	Day 3	Day 21	5^th^ week	5	6
6	mChitosan(OMP+FLA)/FLA -NP WCL 10 + Challenge	Day 3	Day 21	5^th^ week	6	6
7	mChitosan (OMP+FLA)/FLA -NP WCL 50 + Challenge	Day 3	Day 21	5^th^ week	6	6
8	mChitosan (OMP+FLA)/FLA -NP GMP 2.5 + Challenge	Day 3	Day 21	5^th^ week	6	6
9	mChitosan (OMP+FLA)/FLA -NP GMP 10 + Challenge	Day 3	Day 21	5^th^ week	5	5
10	mChitosan (OMP+FLA)/FLA -NP GMP 50 + Challenge	Day 3	Day 21	5^th^ week	6	7

NA, Not applicable; DPC, Days post challenge.

### Bacterial enumeration

To determine the challenge SE load in chickens, the cecum was aseptically collected in Whirl-Pak bags during necropsy, and the bacterial load was estimated as described previously (7). Briefly, the cecal content was weighed and diluted 1:5 (w/v) in PBS according to their weight. Furthermore, ten-fold serial dilutions were made and plated in duplicate on Xylose Lysine Tergitol 4 (XLT4) agar plates containing the antibiotics nalidixic acid and novobiocin, both at 20 µg/mL. The plates were incubated at 37°C for 24 h, and the black colonies were counted. The *Salmonella* counts were recorded as CFU present in the cecal content, and the data were converted to log CFU/g for statistical analysis. Samples negative on direct plating were enriched in tetrathionate broth by incubating at 37°C for 24 h and then plated on XLT4 agar plate. Samples positive on enrichment were considered for determination of CFU and were given a limit of detection value of the direct plating which was 500.

### Antibody ELISA

We measured OMP- and FLA-specific secretory (s) IgA production in cloacal swab specimen, bile and small intestinal wash, and IgG antibodies in serum and bile samples by enzyme-linked immunosorbent assay (ELISA) as previously described (10). Briefly, 96-well ELISA plates (Greiner bio-one, Monroe, NC, USA) were coated overnight in coating buffer in duplicate wells with pre-titrated amounts of OMP and FLA separately at 50 ng/well for IgG determination and OMP at 50 ng and FLA at 400 ng/well for sIgA determination. The plates were washed three times in PBS-T [1× PBS with 0.05% Tween-20 (Sigma-Aldrich)], and the non-specific binding sites were blocked using 5% non-fat dry milk in PBS-T at 200 µL/well for 1 h at room temperature (RT). The samples were diluted in 2.5% dry milk in PBS-T and incubated at 37℃ for 2 h. The specific secondary antibody, goat anti-chicken IgA peroxidase-labeled (Biorad, Kidkington, UK) or goat anti-chicken IgY (H+L) peroxidase-labeled (Southern Biotech, Birmingham, AL, USA) at pre-titrated dilution and diluted in 2.5% dry milk powder in PBS-T were added and incubated at room temperature for 2 h. Following three washes with PBS-T, 50 µL/well of a 1:1 mixture of peroxidase substrate solution B and 3,3′,5,5′-tetramethylbenzidine (Thermo Fisher Scientific) was added. The plates were developed in the dark, and the reaction was halted by adding 50 µL/well of 1M phosphoric acid. The signal was read at OD450 nm using a microplate reader (SpectramaxPlus, Molecular Devices, Sunnyvale, CA, USA). The final OD was calculated by subtracting the average blank readings from the average readings of the duplicate test samples.

### Ribonucleic acid isolation from cecal tonsils

The cecal tonsils were collected in RNAlater™ solution (Invitrogen by Thermo Fisher Scientific, Vilnius, Lithuania) and stored at -80°C until processed. A small part of the cecal tonsils was homogenized by adding Triazol^R^ reagent (Invitrogen by Thermo Fisher Scientific). Chloroform at 200 μL was added, mixed, and incubated for 8 min. Subsequently, the mixture was centrifuged to achieve phase separation. Following centrifugation, the upper aqueous phase containing RNA was collected and precipitated using isopropanol, washed twice with ethanol, and dissolved in RNase-free water. The purity of the RNA was assessed using the NanoDrop spectrophotometer (Thermo Scientific, Waltham, MA, USA) by measuring the ratio of absorbance at 260/280 and 260/230 nm.

### Quantitative real-time PCR

A total of 2,000 ng extracted RNA was used for the cDNA synthesis via reverse transcription template in a 20-μL reaction volume containing the reaction buffer as described previously (7). Briefly, the RNA was denatured at 70°C for 12 min using a thermocycler. Each reaction of c-DNA conversion contains 4 µL of M-MLV RT 5× buffer, 1 µL of 10 mM dNTPs, 0.5 µg of Oligo dT 15 primer, 100 units of M-LV reverse transcriptase, 8 units of recombinant RNAasin ribonuclease inhibitor (Promega, Madison, WI, USA), and 2 µL of 0.1M DTT (Invitrogen by Thermofisher Scientific, Carlsbad, CA, USA). The cytokine gene mRNA expression was quantified by SYBR green method using Quantstudio 5 (Applied Biosystems by Thermofisher Scientific). The reaction mixture that comprised 10 parts of PerfeCTa SYBR Green SuperMix (Quantabio, Beverly, MA, USA), one part of cDNA (50 ng) template, and two parts each of the forward and reverse primers was made up to a final volume of 20 μL with RNAse-free water. The amplification protocol began with initial denaturation at 95°C for 5 min (1 cycle), proceeding with 40 cycles of 94°C for 20 s and the respective annealing temperature for 32 seconds (**Supplementary Table S1**). This was followed by sequential steps at 95°C for 15 s, 60°C for 1 min, 95°C for 15 s, and 60°C for 15 s. Housekeeping gene β-actin was used as the reference gene to normalize Ct values. All data were adjusted to mRNA levels of the mock group and expressed as fold-change using the 2^−ΔΔCt^ method as described previously (19–22).

### Flow cytometry

Splenocytes isolated on the day of necropsy from all the birds were subjected to flow cytometry as described previously (7). Briefly, 5 million cells in 1 mL complete RPMI medium were seeded per well in a 48-well flat bottom plate and stimulated with pooled equal amounts of OMP and FLA (10 μg/mL) for 48 h. The cells (1 × 10^6^) were transferred to a non-sterile 96-well round-bottom FACS plate, washed (centrifugation at 2,000 rpm for 2 min) once in 200 µL FACS buffer/well, and blocked for Fc receptors by using a blocking buffer (1% normal mouse serum in FACS buffer) for 30 min at 4°C. After two washes with 1X PBS, live/dead cell staining was performed using the Fixable Dead Cell Stain Kit (Invitrogen, ThermoFisher Scientific) at 1:1,000 dilution in PBS, with 50 µL/well for 30 min at 4°C. The cells were washed once in FACS buffer and proceeded for surface immunostaining using the anti-chicken lymphocyte-specific antibodies or their corresponding isotype control antibodies at previously optimized concentrations in a final volume of 50 µL/well for 30 min at 4°C (**Supplementary Tables S2, S3**). Samples were acquired using a live cell gating on a BD FACS Aria II machine, and data were analyzed using the FlowJo software (BD Life Sciences).

### Statistical analysis

Statistical differences among groups were determined by one-way ANOVA followed by Tukey’s multiple-comparison test using Prism 10 (GraphPad Software, Inc., CA, USA).

## Results

### Characterization of mannose-chitosan-nanoparticle-based *Salmonella* subunit vaccine

The quality of the proteins used in the mChitosan (OMP+FLA)/FLA-NP vaccine formulation was assessed using SDS-PAGE. The major bands for OMP were observed at expected molecular weights of around 52, 37, and 28 kDa. For Flagella, a major band of protein was detected at approximately 52 kDa (**Supplementary Figure S1**).

The physical nature of mChitosan-NP entrapped with vaccine antigens (OMP+FLA) and adjuvants (WCL and c-di-GMP) was analyzed by using the Malvern Zetsizer to determine the size, polydispersity index, and zeta potential. Furthermore, we estimated the encapsulation efficiency of antigens and adjuvants in the mChitosan-NP formulations (**Table 2**). Our data indicated that all the three analyzed physical natures of the three NP formulations were slightly variable compared to the empty NPs, with high positive charge ranging value of Zeta potential +20-25mV, size distribution 235-260nm, and polydispersity index 0.35-0.52 (**Table 2**). This variability is attributed to the type of cargo entrapped in mChitosan-NP formulations.

**Table 2:** The physical nature of mChitosan-NP entrapped with Salmonella antigens or adjuvants determined using the Malvern Zetasizer.

	Size (nm)	Polydispersity index	Zeta potential (+mV)	Encapsulation efficiency %
**Empty mChitosan-NP**	162	0.2595	+37mV	–
**mChitosan (OMP+FLA)/FLA-NP**	239.6	0.3575	+21mV	70.11%
**mChitosan (GMP)/FLA-NP**	267.2	0.519	+25.62mV	60%
**mChitosan (WCL)/FLA-NP**	254	0.4629	+20.1mV	64.42%

mChitosan, mannose conjugated chitosan; (OMP+FLA)/FLA, Outer membrane proteins and Flagella of Salmonella Enteritidis entrapped in mChitosan nanoparticle and surface coated with flagella; NP, nanoparticle; WCL, Whole cell lysate of Mycobacterium smegmatis; GMP, Cyclic-di-Guanosine Monophosphate; Ch, Challenge.

### Antibody responses against *Salmonella* antigens post-vaccination before challenge

The chickens received two doses of candidate vaccine and adjuvant combinations; the prime dose was at 3 days of age and the booster at 21 days (**Figure 1**). The commercial vaccine was administered on days 1 and 14 of age as per the manufacturer’s guidelines. To assess OMP- and FLA-specific IgY and sIgA levels in vaccinates at post-vaccination and pre-challenge stage, serum and cloacal swabs were collected from vaccinated birds prior to administering the booster dose (pre-booster) and 2 weeks after the booster inoculation at 1 day before challenge infection (post-booster).

**Figure 2:**
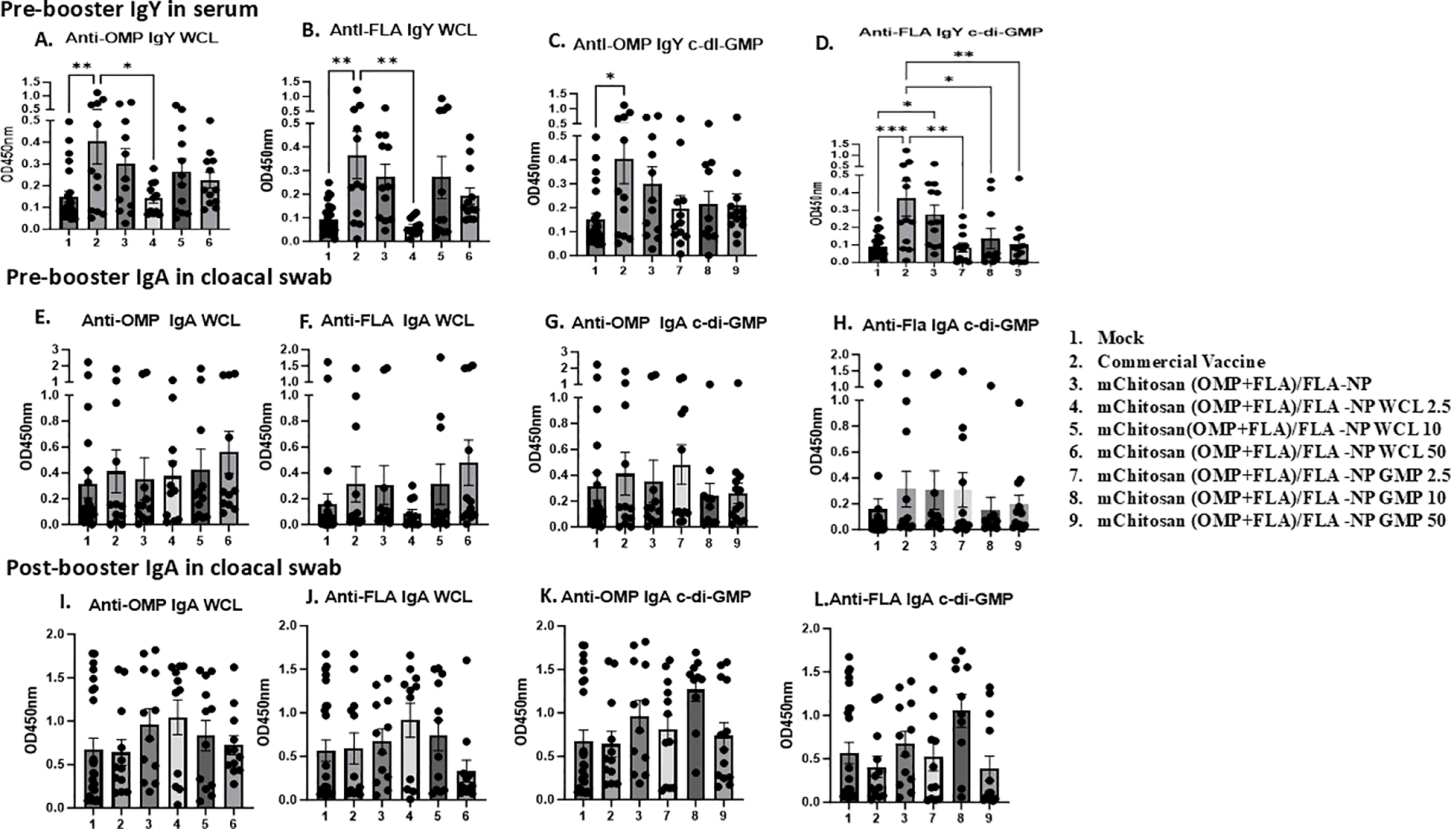
Systemic and mucosal *S.* Enteritidis OMP- and FLA-specific IgY and sIgA antibody responses in broilers inoculated orally with adjuvanted mChitosan (OMP+FLA)/FLA-NP vaccine. The serum samples collected at pre-booster stage **(A–D)** and the cloacal swabs collected at pre-booster **(E–H)** and post-booster stages **(I–L)** were analyzed by ELISA. Each bar is the mean ± standard error of mean (SEM) of 10–13 birds per group. The *P*-values between groups were determined by one-way ANOVA, followed by Tukey’s multiple-comparisons *post-hoc* test. *p < 0.05; **p < 0.01; ***p < 0.001.

In pre-booster samples, an increased trend in IgY levels in mChitosan (OMP+FLA)/FLA-NP and mChitosan (OMP+FLA)/FLA-NP WCL 10-µg vaccine groups against both OMP and FLA proteins compared to the control groups was observed, while in the group that received commercial vaccine, a significantly higher IgY against both OMP and FLA was observed (**Figures 2A, B**), but no such increase was detected in mChitosan (OMP+FLA)/FLA-NP GMP groups at pre-booster both in serum and in cloacal swabs (**Figures 2C–H**). In post-booster cloacal swabs, only in 2.5 µg mChitosan (OMP+FLA)/FLA-NP WCL and 10 µg mChitosan (OMP+FLA)/FLA-NP GMP vaccinated groups was a numerical increase in sIgA against OMP and FLA proteins detected compared to the rest of the experimental groups (**Figures 2I–L**).

### Antibody responses against *Salmonella* antigens in vaccinated birds post-challenge

**Figure 3:**
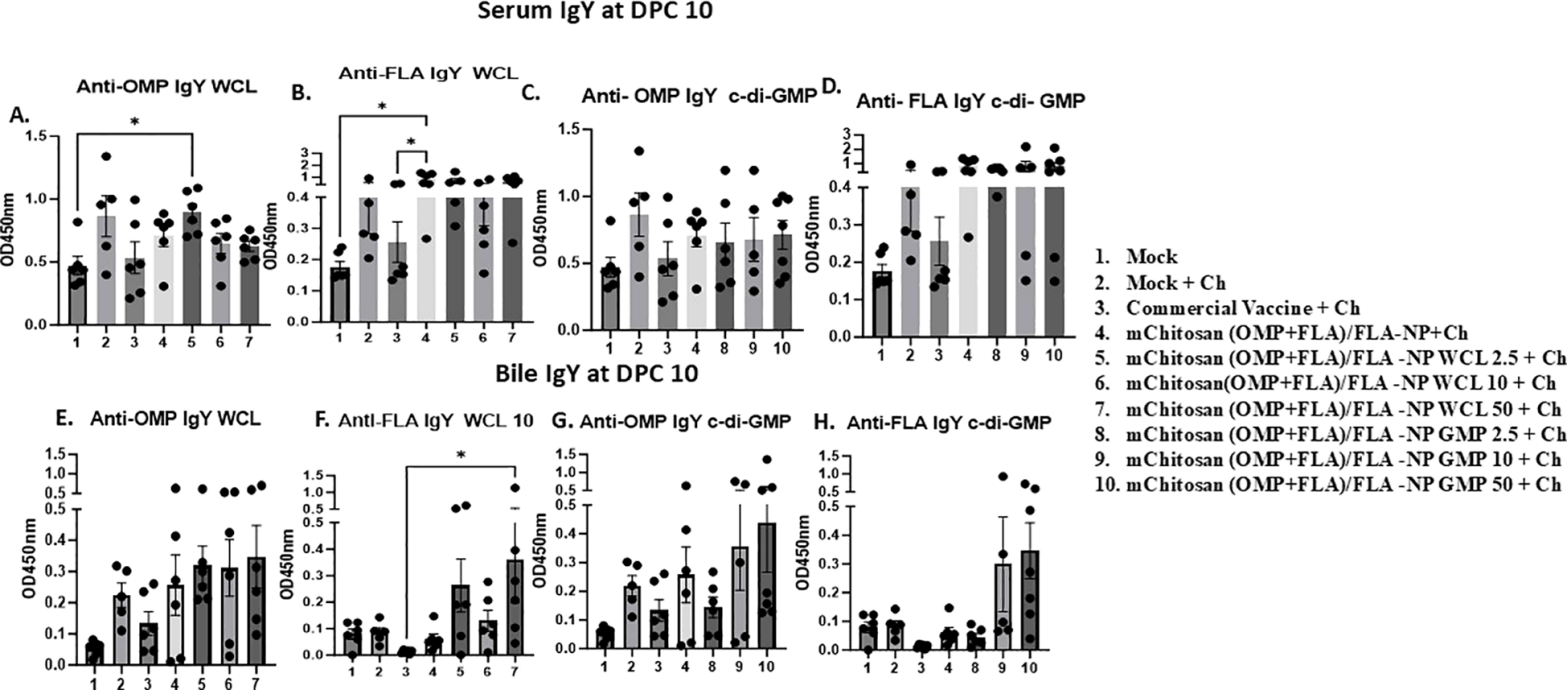
Systemic *S*. Enteritidis OMP- and FLA-specific IgY antibody response in broilers inoculated orally with adjuvanted mChitosan (OMP+FLA)/FLA-NP vaccine. The samples collected at DPC10 were analyzed by ELISA in serum **(A–D)** and bile **(E–H)**. Each bar is the mean ± SEM of five to seven birds per group. The *P*-values between groups were determined by one-way ANOVA, followed by Tukey’s multiple-comparisons *post-hoc* test. *p < 0.05.

IgY levels: At DPC4, the antigen-specific IgY levels in the serum of vaccinated groups did not increase substantially over the pre-challenge levels. However, in the bile, OMP-specific IgY was observed at levels slightly higher than in the commercial vaccine, mChitosan (OMP+FLA)/FLA-NP WCL, and GMP 50 µg/dose groups. In addition, the flagella-specific IgY level was relatively higher compared to the other groups in the mChitosan (OMP+FLA)/FLA-NP GMP 50-µg/dose vaccine group (data not shown). By DPC10, the IgY antibody response was substantially increased in all of the vaccinated groups, particularly against FLA in both serum and bile (**Figure 3**). In particular, mChitosan (OMP+FLA)/FLA-NP WCL 2.5-µg/dose elicited relatively higher IgY levels against OMP in serum. Interestingly, the mChitosan (OMP+FLA)/FLA-NP (administered without any adjuvant) group had significantly higher levels of IgY response against FLA antigen compared to the mock and commercial vaccine groups (*p* < 0.05) (**Figure 3B**), while in the bile, the flagella-specific IgY level was significantly (*p*< 0.05) higher in mChitosan(OMP+FLA)/FLA-NP WCl-50µg compared to the commercial vaccine group (**Figure 3F**).

**Figure 4:**
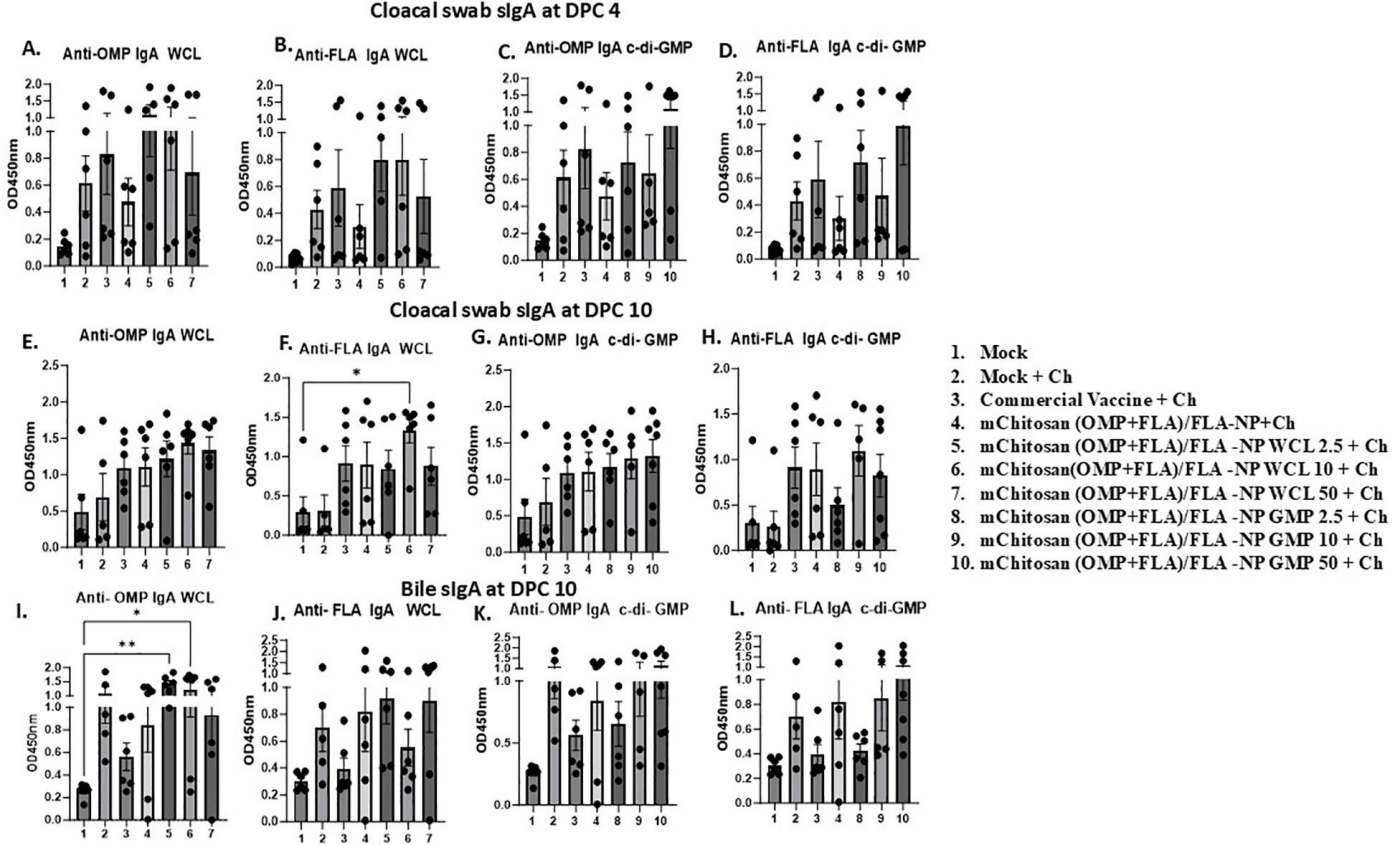
Mucosal *S*. Enteritidis OMP- and FLA-specific sIgA antibody responses in broilers inoculated orally with adjuvanted mChitosan (OMP+FLA)/FLA-NP vaccine. The cloacal swabs collected at DPC 4 **(A–D)** and DPC10 **(E–H)** and the bile samples collected at DPC10 **(I–L)** were analyzed by ELISA. Each bar is the mean ± SEM of five to seven birds per group. The *P*-values between groups were determined by one-way ANOVA, followed by Tukey’s multiple-comparisons *post-hoc* test. *p < 0.05; **p < 0.01.

sIgA levels: Specific sIgA antibody responses against OMP and FLA post-challenge was evaluated in cloacal swab and bile samples. At DPC4, mChitosan (OMP+FLA)/FLA-NP WCL at 2.5 and 10 µg/dose and mChitosan (OMP+FLA)/FLA-NP GMP at 50 µg/dose had relatively higher sIgA levels in the cloacal swab against both OMP and FLA antigens compared to the other groups (**Figures 4A–D**). However, the sIgA levels in the bile was not notably different among all of the vaccinated groups at DPC4 (data not shown), while the specific sIgA response was highly pronounced at DPC10 in the mChitosan (OMP+FLA)/FLA-NP WCL 10-µg/dose group against FLA (**Figure 4F**) and, particularly against FLA protein, was significantly higher compared to the mock group (*p* < 0.05) (**Figure 4F**). Meanwhile, in the c-di-GMP adjuvanted vaccine groups, the level of sIgA in the cloacal swab was only slightly higher, and the data were not statistically significant (**Figures 4G, H**).

In bile, both the mChitosan (OMP+FLA)/FLA-NP WCL 2.5- and 10-µg/dose groups elicited significantly higher OMP-specific sIgA titers compared to the mock group (*p* < 0.01 and *p* < 0.05) (**Figure 4I**), and a consistently increasing FLA-specific trend was detected (**Figure 4J**). From among the three c-di-GMP groups, both mChitosan (OMP+FLA)/FLA-NP GMP 10- and 50-µg/dose vaccine receiving groups showed relatively higher titers of sIgA against OMP and FLA proteins (**Figures 4K, L**).

Although sIgA levels were also measured in the small intestine, no significant differences were observed among the vaccine groups (data not shown). It is important to note that although the specific sIgA titers in both cloacal swab and bile were low at DPC4, they increased substantially by DPC10 (**Figures 4E–L** and data not shown).

### Quantification of cytokine gene expression in the cecal tonsils

**Figure 5:**
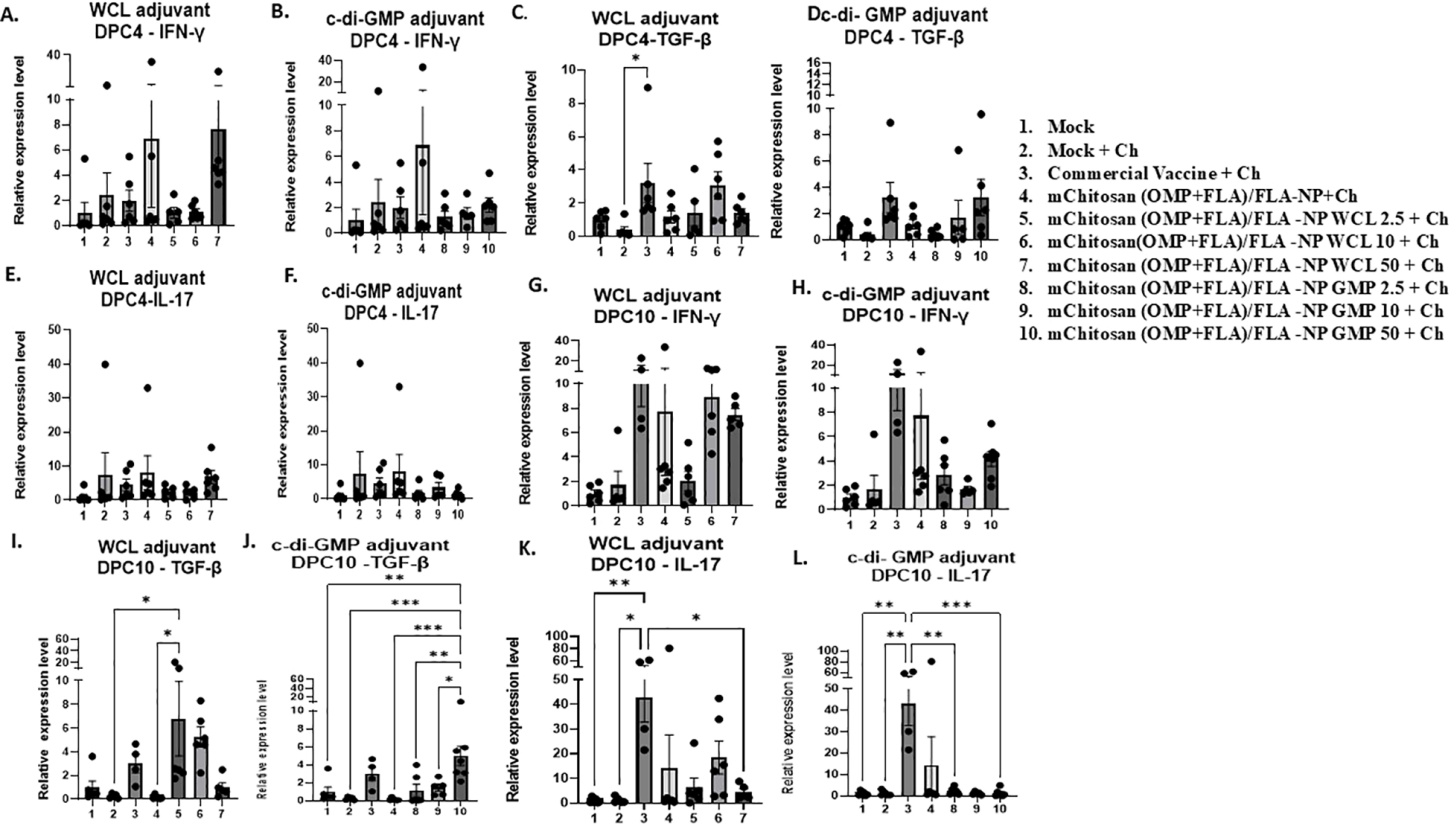
Cytokine gene expression analysis in the cecal tonsils of broilers inoculated orally with adjuvanted mChitosan (OMP+FLA)/FLA-NP vaccine at DPC4 and DPC10. IFN-γ **(A, B, G, H)**, TGF-β **(C, D, I, J)** and IL-17 **(E, F, K, L)** gene expression. Each bar is the mean ± SEM of fold change in the gene expression of five to seven birds per group. The *P*-values between groups were determined by one-way ANOVA, followed by Tukey’s multiple-comparisons *post-hoc* test. *p < 0.05; **p < 0.01; ***p < 0.001.

We observed upregulation of the IFN-γ gene expression in mChitosan (OMP+FLA)/FLA-NP WCL 50-µg/dose and mChitosan (OMP+FLA)/FLA-NP without any adjuvant groups at DPC4 (**Figure 5A**). However, no upregulation was observed in c-di-GMP groups (**Figure 5b**). The TGF-β expression was significantly higher in the commercial vaccine group compared to the mock challenge group (*p* < 0.05), along with relative upregulation in mChitosan (OMP+FLA)/FLA-NP WCL 10-µg/dose and mChitosan (OMP+FLA)/FLA-NP GMP 50-µg/dose groups compared to the other groups (**Figures 5C, D**). At DPC10, there was an increasing trend in IFN-γ gene expression in mChitosan (OMP+FLA)/FLA-NP, mChitosan (OMP+FLA)/FLA-NP WCL 10 and 50 µg/dose, mChitosan (OMP+FLA)/FLA-NP GMP 50 µg/dose, and commercial vaccine groups compared to the mock and mock challenge groups (**Figures 5G, H**). The TGF-β expression in mChitosan (OMP+FLA)/FLA-NP WCL 2.5-µg/dose group was significantly higher compared to that of the mock challenge and mChitosan (OMP+FLA)/FLA-NP without adjuvant groups (*p* < 0.05) (**Figure 5I**). The expression of TGF-β in mChitosan (OMP+FLA)/FLA-NP GMP 50-µg/dose vaccine group was significantly higher than the rest of the vaccine combination groups (**Figure 5J**). The cytokine IL-17 gene expression was significantly upregulated only in the commercial vaccine group at DPC10 (**Figures 5E, F, K, L**).

### Modulation of B and T-cell frequency in mChitosan (OMP+FLA)/FLA-NP vaccinates

**Figure 6:**
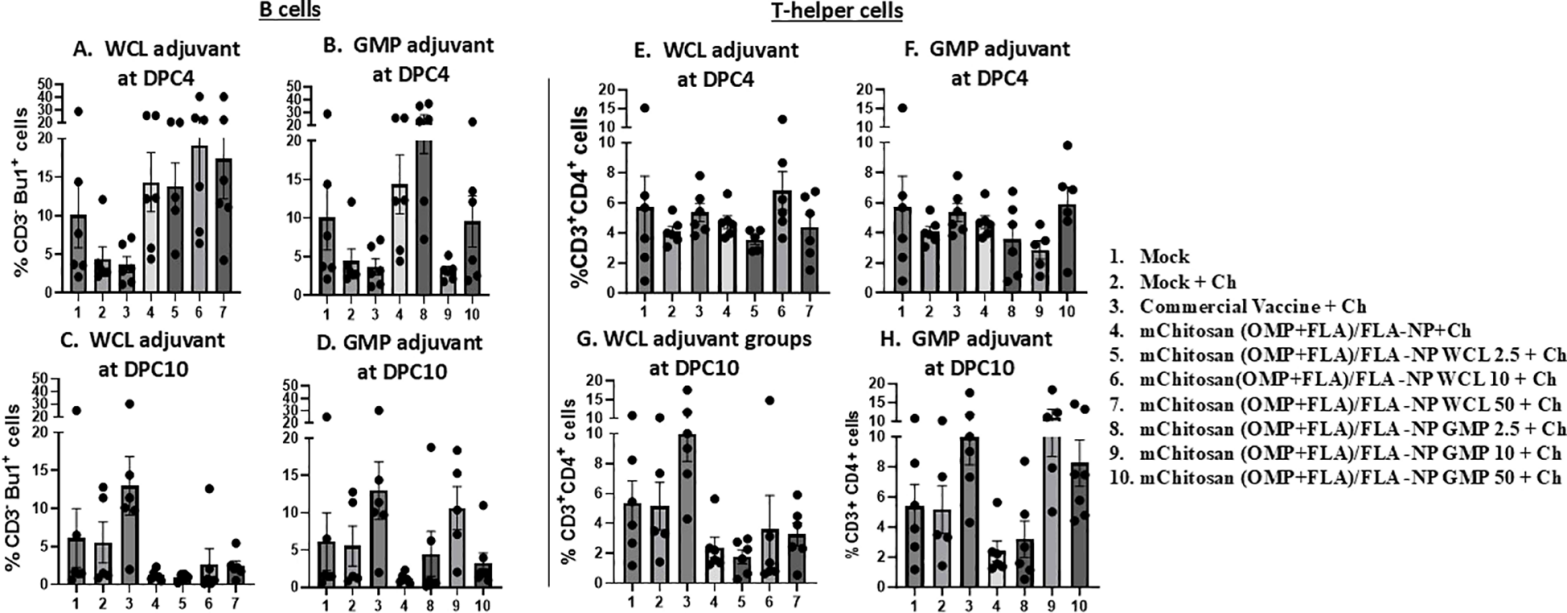
Frequency of recall lymphocytes’ response in the spleen. The birds were inoculated orally with adjuvanted mChitosan (OMP+FLA)/FLA-NP vaccine. The splenocytes isolated at DPC4 and DPC10 were stimulated with OMP+FLA antigens for 48 h, immunostained using chicken-lymphocyte-specific monoclonal antibodies, and subjected to flow cytometry analysis. Frequency of **(A–D)** B cells (CD3^-^Bu1^+^) and **(E–H)** T-helper cells (CD3^+^CD4^+^). Each bar is the mean ± SEM of percent B cells and T-helper cells of five to seven birds per group. The *P*-values between groups were determined by one-way ANOVA, followed by Tukey’s multiple-comparisons *post-hoc* test.

Flow cytometry analysis was performed to assess the frequency of major lymphocyte subsets such as TCRγδ, CD4 and CD8 T cells, and B cells in a recall immune response analysis of splenocytes. The frequency of B cells (CD3^-^BU1^+^) increased numerically in mChitosan (OMP+FLA)/FLA-NP WCL 2.5, 10, and 50 µg/dose and GMP 2.5 µg/dose and without any adjuvant vaccinated groups at DPC4 (**Figures 6A, B**), which was in a decreasing trend in all of those vaccinated groups by DPC10 (**Figures 6C, D**). Meanwhile, the frequency of B cells in the commercial vaccine group was exactly in the opposite trend with increased population at DPC10 and reduced at DPC4 (**Figures 6A–D**).

At DPC4, in none of the mChitosan (OMP+FLA)/FLA-NP vaccinates and commercial vaccine group did the frequency of T-helper cells (CD3^+^CD4^+^) had any changes (**Figures 6E, F**). In contrast, at DPC10, the T-helper cell population was increased in mChitosan (OMP+FLA)/FLA-NP GMP 10- and 50-µg/dose and commercial vaccine receiving groups, while none in the WCL adjuvanted mChitosan (OMP+FLA)/FLA-NP vaccine groups (**Figures 6G, H**). The frequency of cytotoxic T cells and TCRγδ cells, respectively, were also assessed in stimulated splenocytes, but there was no substantial difference among the vaccinated groups at both DPC4 and 10 (data not shown).

### Challenge bacterial load in the cecum of mChitosan (OMP+FLA)/FLA-NP vaccinates

All of the bird groups challenged with *S.* Enteritidis were evaluated for cecal bacterial load at both DPC4 and 10. As expected, the mock no challenge group was negative for SE, while at DPC4, the mock challenge group had an average of 4 log_10_ CFU/g SE load in the cecum. Among the three different doses of WCL adjuvanted mChitosan (OMP+FLA)/FLA-NP vaccinated groups, we observed a greatest reduction in the challenge SE load in the WCL 10-µg/dose group with 0.53 log_10_ reduction at DPC4, which remained consistently reduced at DPC10 with 0.61 log_10_ reduction compared to the mock challenge group. This level of bacterial reduction in the cecum was comparable to the commercial vaccine receiving group (**Table 3**). Among the GMP-adjuvanted vaccine groups, mChitosan (OMP+FLA)/FLA-NP GMP 50 µg/dose induced the greatest reduction of 1.2 log_10_ in the cecal bacterial load, but by DPC10, there was absence of any reduction compared to the mock challenge group (**Table 3**). However, there was no apparent bacterial reduction in the group that received the vaccine without any adjuvant. In addition, mChitosan (OMP+FLA)/FLA-NP GMP 2.5 µg/dose induced 0.37 and 0.53 log_10_ reduction in the bacterial load at DPC4 and 10, respectively (**Table 3**).

**Table 3:** Reduction in challenge S. Enteritidis load in the cecum of broilers inoculated orally with adjuvanted mChitosan (OMP+Fla) vaccine compared to the mock challenge.

Experiment vaccine groups (n=5-7 birds per group)	Mean± SE Log_10_ CFU/ml at DPC4	Mean± SE Log_10_ CFU/ml at DPC10	Log_10_ reduction compared to mock challenge at	
			DPC4	DPC10
**Mock + Challenge**	3.98 ±0.46	2.86±0.75		
**Commercial live Salmonella vaccine + Ch**	3.47±0.35	2.11±0.73	0.51	0.75
**mChitosan (OMP+FLA)/FLA-NP + Ch**	4.08±0.49	3.03±0.28	0	0
**mChitosan (OMP+FLA)/FLA-NP WCL 2.5 + Ch**	3.76±0.13	3.54±0.30	0.22	0
**mChitosan(OMP+FLA)/FLA -NP WCL 10 + Ch**	3.45±0.32	2.25±0.450	0.53	0.61
**mChitosan (OMP+FLA)/FLA-NP WCL 50 + Ch**	3.63±0.41	2.77±0.57	0.35	0.09
**mChitosan (OMP+FLA)/FLA-NP GMP 2.5 + Ch**	3.61±0.33	2.33±0.47	0.37	0.53
**mChitosan (OMP+FLA)/FLA-NP GMP 10 + Ch**	3.56±0.23	2.98±0.28	0.42	0
**mChitosan (OMP+FLA)/FLA-NP GMP 50 + Ch**	2.77±0.64	3.32±0.29	1.21	0

Each value is the mean +/- SE of 5-7 birds per group.

mChitosan, mannose conjugated chitosan; (OMP+FLA)/FLA, Outer membrane proteins and Flagella of Salmonella Enteritidis entrapped in mChitosan nanoparticle and surface coated with flagella; NP, nanoparticle; WCL, Whole cell lysate of Mycobacterium smegmatis; GMP, Cyclic-di-Guanosine Monophosphate; Ch, Challenge.

## Discussion

**Figure 7:**
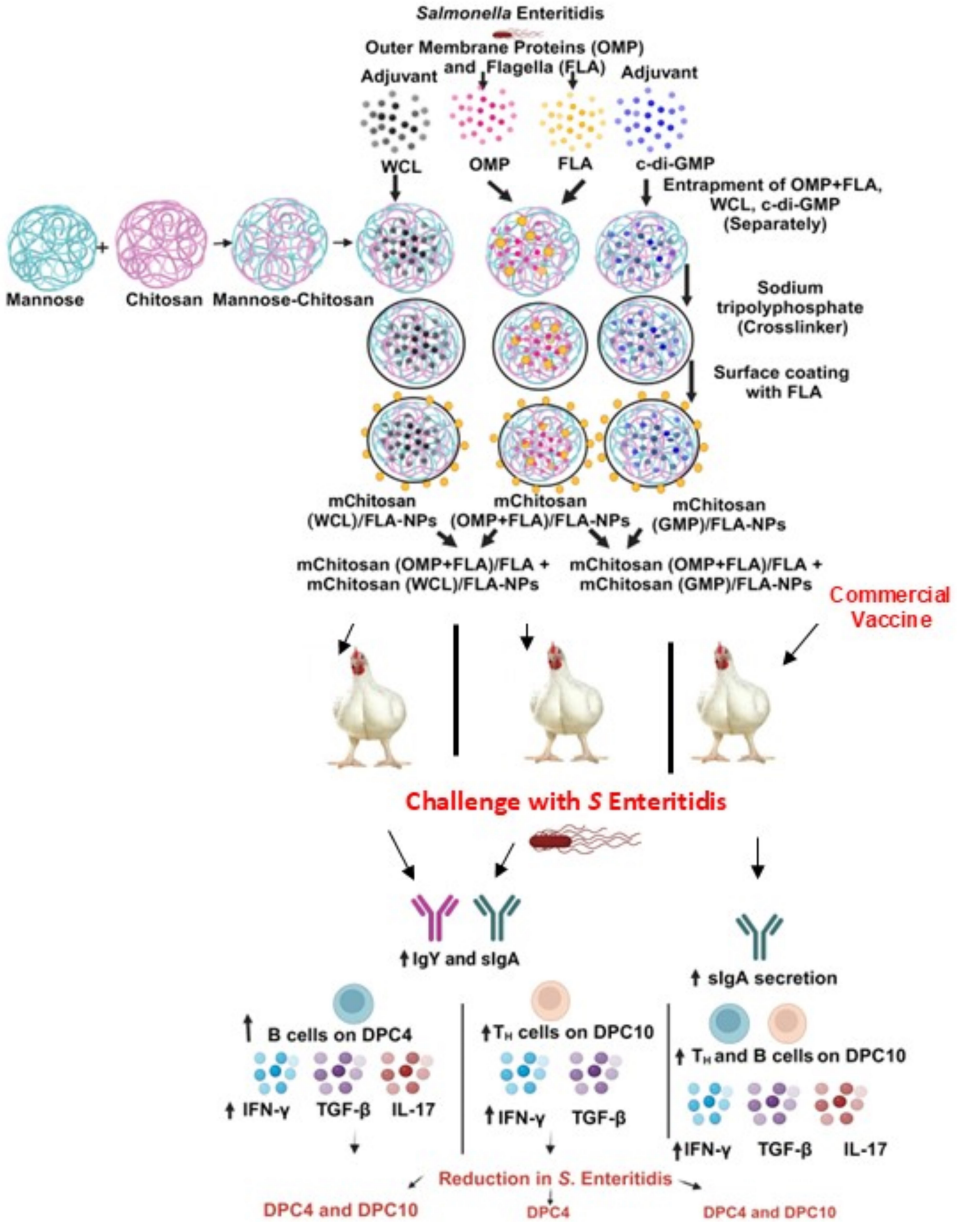
A schematic of the complete overview of mChitosan (OMP+FLA)/FLA-NP vaccine and adjuvant combination and commercial live *Salmonella* vaccine outcomes in orally vaccinated and challenge birds. Created with BioRender.com

In this study, we evaluated the efficacy of a fixed dose (20 µg) of combined OMP+FLA *Salmonella* antigens entrapped in mChitosan-NP coadministered with three different doses of two potent mucosal adjuvants (c-di-GMP and WCL from *M. smegmatis*), which were also entrapped in mChitosan-NP against the colonization of *S.* Enteritidis in broilers (**Figure 7**). In a previous Chitosan-NP vaccine trial, we evaluated the efficacy of two and three doses of 10 and 50 µg/dose of combined OMP+FLA *Salmonella* antigens vaccinated orally without any adjuvant in broilers (10). In this mChitosan (OMP+FLA)/FLA-NP vaccine trial, our goal was to potentiate the level of immunity in the intestines of birds; therefore, we evaluated the adjuvant dose response. It is important to initially assess different doses of mucosal adjuvant to prevent potential tolerance to a vaccine in animals and at the same time ensure the induction of protective immune responses.

In addition, antigen dose also influences the immune response. Unlike in our earlier study in broiler chickens (10), our goal in this study was to achieve a balanced *Salmonella*-specific Th1 and Th2 immune response to mChitosan (OMP+FLA)/FLA-NP vaccine. Therefore, we used 20 µg of pooled OMP+FLA antigen in each vaccine dose. Previous studies have shown that a low antigen load tends to favor a T-helper type 2 (Th2) cytokine response, while a high antigen load favors a Th1 cytokine response (23). Additionally, other studies have shown that antigen dosing can have important implications, often representing a delicate balance between tolerance and induction of protective immunity (14, 15). A vaccine study used SARS-CoV-2 Spike protein administered repeatedly, and in another study, a high dose of antigen in a tuberculosis vaccine trial was found detrimental for protection, mediated by T cell exhaustion and immune tolerance (14, 15). We found that a lower dose of 10 μg of *Salmonella* subunit antigens *versus* a dose of 50 μg delivered in Chitosan-NP orally augments both mucosal and systemic immune responses, resulting in a comparable reduced bacterial load in the intestines of birds (7). Therefore, considering the potent mucosal adjuvanticity of WCL and GMP observed in this study to mChitosan (OMP+FLA)/FLA-NP oral vaccine (**Figure 7**), in future vaccine trials, we will use an optimum single dose of the adjuvant (WCL 10 μg/dose) with lower doses of *Salmonella* subunit antigens (1, 5, and 10 μg) delivered in mChitosan-NP.

The quality of OMP and flagella proteins used in this study was assessed by SDS-PAGE analysis by comparing them with the previously extracted proteins used *in vivo* (7, 10), and we found them satisfactory with three major bands at 52, 37, and 28 kDa (17, 24–26). The immunodominant antigen OMP elicits adaptive immune responses against salmonellosis by activating dendritic cells (27). Dendritic cells can recognize pathogen-associated molecular patterns from bacteria and then present antigen to naïve T cells in the secondary lymphoid organs through major histocompatibility complex (MHC) class I and II molecules. Among the 22 species of OMP, OmpA is the most abundant protein with size ranging from 28 to 38 kDa. OmpA is also known to stimulate a strong antibody response (28). The other antigen used in the vaccine was flagella, and its major band was observed at approximately 52 kDa, which was consistent with a previous study (29). Flagella is one of the pathogen-associated molecular patterns recognized by Toll like receptor-5. Binding of flagella to PAMPs results in the production of pro-inflammatory cytokines (30). Flagella is a virulence factor involved in the invasion and attachment of bacteria in the small intestines, especially in the ileum (31, 32), which is rich in immune inductive lymphoid tissues called Peyer’s patches.

While OMP and flagella antigens possess immunostimulatory properties, they lack the ability to effectively reach the intended immune sites when delivered orally in their native soluble form. Therefore, we utilized chitosan-NP as a carrier for the vaccine cargo. Chitosan is a biopolymer obtained from the exoskeleton of crustaceans (33). It is a cationic polysaccharide consisting of two units of d-glucosamine and N-acetyl-d-glucosamine. It has a strong positive charge and is soluble in weak acidic solution (34, 35). Our vaccine NPs carry a strong positive charge (+20 to +25 mV), contributed by amines in the chitosan, helping in the interaction with the sialic acid residues of glycoproteins in the mucus and epithelial cells that carry a strong negative charge on the surface. This interaction enhances the exposure time of chitosan-NP with the mucus membrane, facilitating the efficient uptake of orally delivered vaccine antigens and internalization by the antigen-presenting cells (22, 36). This interaction is further enhanced by the binding of mannose conjugated to chitosan-NP with mannose receptors present on monocytes, dendritic cells, and macrophages (37). Furthermore, unique characteristics of flagella have been exploited, wherein mChitosan-NPs were surface-coated with flagella for prolonged localization of the vaccine NPs in the ileum’s immune inductive sites called Peyer’s patches (9, 11).

The size of NPs is a critical parameter for the uptake of antigens by dendritic cells and macrophages. A particle of 20–200-nm size is taken up readily by dendritic cells through clathrin-dependent endocytosis, while they can efficiently internalize particles up to 500 nm. The NPs used in this study were of 235–260 nm in size. The internalized NPs are processed by dendritic cells and present antigen to naïve T cells (38, 39). Polydispersity index (PDI) is another important feature of NP-based vaccines, as it determines the distribution of particle size in the vaccine formulation, representing the degree of heterogeneity or uniformity in particles. The PDI value ranges from 0 (for a perfectly homogenous sample) to 1 (for a heterogenous sample) (40). PDI below <0.5 indicates monodisperse distribution (41). Our mChitosan (OMP+FLA)/FLA-NP formulation had a PDI value below <0.5 and thus was found ideal for vaccination in addition to other favorable features for oral vaccination in birds.

The vaccination schedule in this study was based on our previous experiments, wherein administering one booster dose yielded favorable outcomes with the bacterial load reduction of about 0.5 log (10). In a subsequent trial, mChitosan-NP vaccine reduced the bacterial load by about 1 log in broilers (7). In our effort to improve the vaccine’s efficiency, adjuvants c-di-GMP and WCL were incorporated in this vaccine trial. The adjuvants were also encapsulated in mChitosan-NP to protect them from degradation and for efficient uptake by intestinal immune cells. As noted in **Table 1**, the mChitosan (OMP+FLA)/FLA-NP coadministered with WCL 10 µg/dose and GMP 50 µg/dose exhibited the greatest reduction in the bacterial load. However, by DPC10, there was no reduction in the bacterial load in the higher two doses of GMP-adjuvanted vaccine groups, while WCL at 10 µg/dose was consistent in its adjuvanticity, indicating that the robust innate immune response induced by GMP appears to be responsible for early protection which disappeared by DPC10.

Unlike in our two earlier chitosan-NP vaccine trials (7, 10), in this vaccine trial, the reduced challenge *S.* Enteritidis load in the adjuvanted vaccine formulation was not satisfactory, suggesting the need to perform a dose–response (1, 5, and 10 µg/dose) analysis of OMP+FLA entrapped in mChitosan-NP coadministered with WCL because a higher dose of OMP+FLA (50 µg in an earlier trial and 20 µg in this trial) in the vaccine did not reduce the bacterial load better than the 10 µg/dose of antigens (10). This indicates the possible immune tolerance that happens when excess OMP+FLA antigens are delivered using the mChitosan-NP system. Thus, using an optimum dose which favors a slightly higher Th2 response than Th1 response appears to be ideal for maximum efficacy in achieving a substantial reduction in the challenge SE load in the cecum of broiler chickens. This is important because based on the Quantitative Microbiological Risk Assessment, two log reductions of *Salmonella* would lead to a reduction of over 90% human salmonellosis cases (42).

In our earlier studies in pigs, when a modified live porcine reproductive and respiratory syndrome virus or the inactivated whole virus entrapped in PLGA-NP was coadministered intranasally with WCL of *Mycobacterium tuberculosis*, a robust improvement in vaccine efficacy to a heterologous challenge virus infection was observed, which was associated with enhanced sIgA production and cellular immune responses (43–45). Our study in chickens reiterated the potent adjuvanticity of WCL to NP-based oral vaccines.

In this study, a progressive increase of sIgA production and expression of TGF-β in the cecal tonsils in WCL-adjuvanted vaccinates from DPC4 to DPC10 indicates the persistent activation of the mucosal B cells in chickens. This was consistent with the detection of increased specific sIgA response in cloacal swab samples at the pre-booster stage. However, the specific IgY response in both mucosal-adjuvant-vaccinated groups was less pronounced than sIgA production, highlighting that the oral vaccine primarily induces local sIgA production rather than systemic responses. Overall, mChitosan (OMP+FLA)/FLA-NP GMP 50 µg/dose and mChitosan (OMP+FLA)/FLA-NP WCL 10 µg/dose performed better in most cases, eliciting a heightened antibody response and a reduction in bacterial load. Meanwhile, WCL 10 µg/dose was consistent in its adjuvanticity at both DPC4 and DPC10, but not GMP 50 µg/dose.

For the first time, we estimated the antigen-specific recall B -cell responses in chickens. Our data indicated an increased frequency of B cells, especially in the WCL-adjuvanted vaccine group at DPC4. However, in a commercial live bacterial vaccine group, an increased population of B cells was delayed. In previous studies, adjuvant c-di-GMP coadministered with an antigen elicited enhanced sIgA and IgY responses in mice (46) and effectively induced both mucosal and systemic immune responses by binding to STING (46–48). These results are consistent with our observations in chickens.

TGF-β expression was upregulated in WCL (2.5 and 10 µg/dose) and c-di-GMP (50 µg/dose) adjuvant vaccinated and in commercial live vaccine receiving groups. Additionally, cytokine IL-17 expression was upregulated only in the commercial vaccine group, wherein a live bacterium was used in the vaccine. The upregulation of both TGF-β and IL-17 indicates the activation of Th17 response, where TGF-β, along with other cytokines, helps naïve CD4+ cells differentiate into Th17 cells. The differentiated Th17 cells secrete IL-17 which contributes to protection against pathogens (49). However, in our adjuvanted (especially WCL) vaccine groups, there was a balanced activation of Th1 and Th2 responses. This was indicated by the upregulation of IFN-γ associated with Th1 response (50) and the secretion of mucosal sIgA associated with Th2 response (51) (**Figure 7**).

IFN-γ is a key proinflammatory cytokine upregulated in response to intracellular pathogens like *Salmonella*. It primarily functions by activating macrophages to release other types of proinflammatory cytokines such as TNF-α and IL-6, thereby regulating both innate and adaptive immunity (52). Secretory IgA is a marker of mucosal immune response (53), and TGF-β promotes sIgA secretion in epithelial cells, and it is an important cytokine in the regulation of mucosal immune responses in the intestines (54, 55). This interaction triggers B cells to undergo immunoglobulin class switch recombination and thus produce sIgA. This process is enhanced by cytokines TGF-β, IL-4, IL-10, IL-5, and IL-21 (56). Cells that produce sIgA in the gut need an extended interaction between B cells and dendritic cells in Peyer’s patches facilitated by TGF-β (57). All of these findings strongly suggest that there was a balanced Th1 and Th2 immune response induced by the mucosal adjuvant WCL to oral mChitosan (OMP+FLA)/FLA-NP vaccine in broiler chickens (**Figure 7**).

## Conclusion

This study evaluated the protective efficacy of mChitosan-NP-based *S.* Enteritidis subunit vaccine coadministered orally with potent mucosal adjuvants and compared the efficacy with a commercial live *Salmonella* vaccine (**Figure 7**). The physical characteristics of the vaccine antigens and adjuvant containing mChitosan-NP formulations were found ideal for oral delivery in chickens. Our data indicated that the mChitosan-NP-based *Salmonella* antigens and WCL (10 µg/dose) combination consistently reduced the challenge bacterial load like the commercial live vaccine. The mChitosan-NP-based Salmonella antigens and WCL adjuvant (10 μg/dose) induced enhanced specific sIgA and IgY production, enhanced B cell population in spleen, IFN-g and TGF-β gene expression in the cecal tonsils (**Figure 7**). Overall, this study highlights the importance of coadministering an optimum dose of a potent adjuvant with *Salmonella* subunit antigens entrapped in mChitosan-NPs orally to elicit superior immune response and efficacy in broilers. Further studies are required to optimize the precise dose of *Salmonella* antigens coadministered with the mucosal adjuvant WCL (10 µg/dose) to achieve robust immune responses associated with a maximum reduction in the colonization of *S*. Enteritidis in the intestines of broilers.

## Data Availability

The raw data supporting the conclusions of this article will be made available by the authors, without undue reservation.
