# Supplementary material for: Evaluation of mucosal adjuvants to chitosan-nanoparticle-based oral subunit vaccine for controlling salmonellosis in broilers
Source: Front Immunol. 2025 Feb 3;16:1509990. doi: 10.3389/fimmu.2025.1509990 (PMC11840259; doi:10.3389/fimmu.2025.1509990)
Supplement: Supplementary file 1 [file SupplementaryFile1.pptx]

## Slide 1
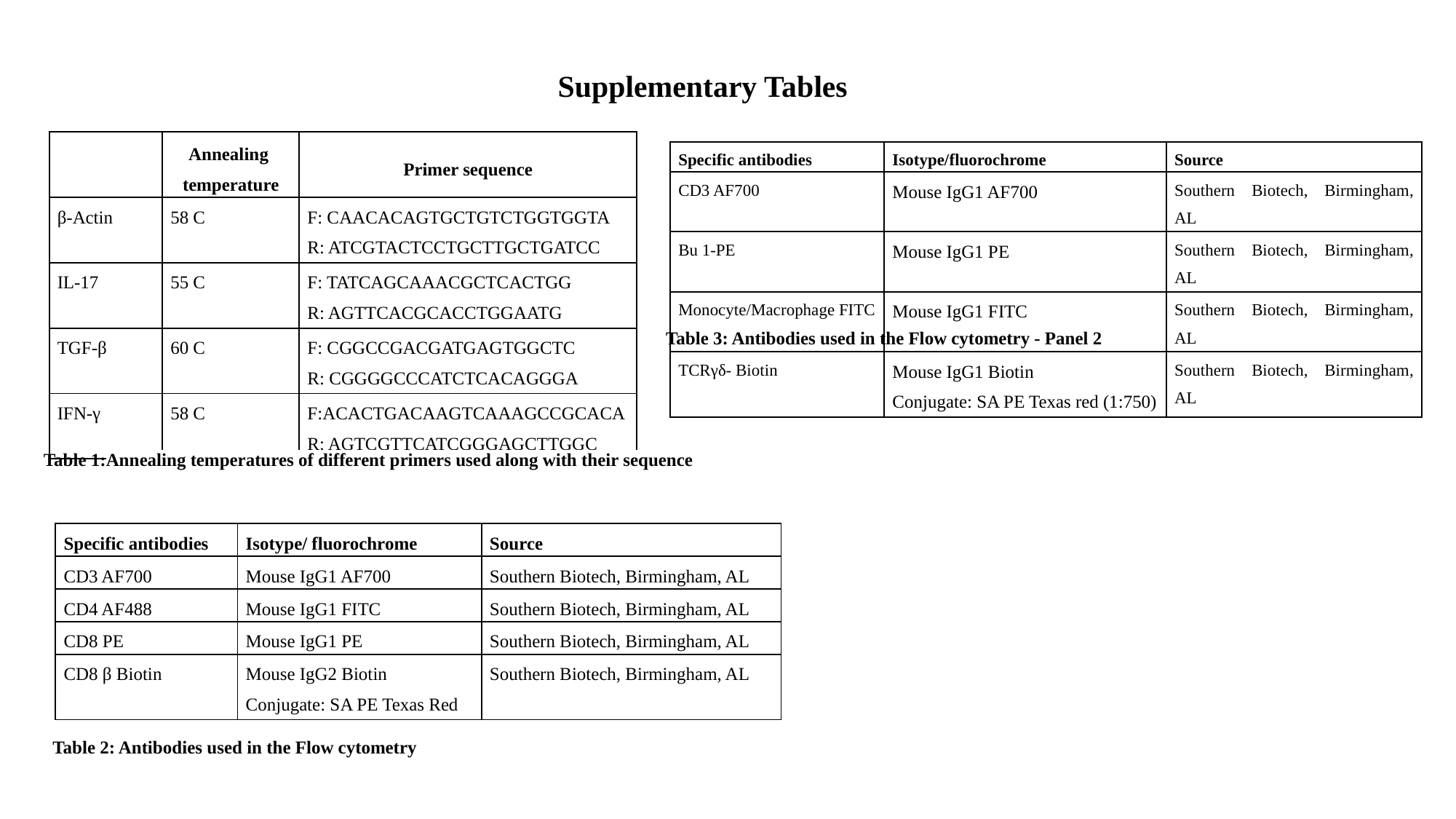

Supplementary Tables
| | Annealing temperature | Primer sequence |
| --- | --- | --- |
| β-Actin | 58 C | F: CAACACAGTGCTGTCTGGTGGTA R: ATCGTACTCCTGCTTGCTGATCC |
| IL-17 | 55 C | F: TATCAGCAAACGCTCACTGG R: AGTTCACGCACCTGGAATG |
| TGF-β | 60 C | F: CGGCCGACGATGAGTGGCTC R: CGGGGCCCATCTCACAGGGA |
| IFN-γ | 58 C | F:ACACTGACAAGTCAAAGCCGCACA R: AGTCGTTCATCGGGAGCTTGGC |
| Specific antibodies | Isotype/fluorochrome | Source |
| --- | --- | --- |
| CD3 AF700 | Mouse IgG1 AF700 | Southern Biotech, Birmingham, AL |
| Bu 1-PE | Mouse IgG1 PE | Southern Biotech, Birmingham, AL |
| Monocyte/Macrophage FITC | Mouse IgG1 FITC | Southern Biotech, Birmingham, AL |
| TCRγδ- Biotin | Mouse IgG1 Biotin Conjugate: SA PE Texas red (1:750) | Southern Biotech, Birmingham, AL |
Table 3: Antibodies used in the Flow cytometry - Panel 2
Table 1:Annealing temperatures of different primers used along with their sequence
Table 2: Antibodies used in the Flow cytometry

## Slide 2
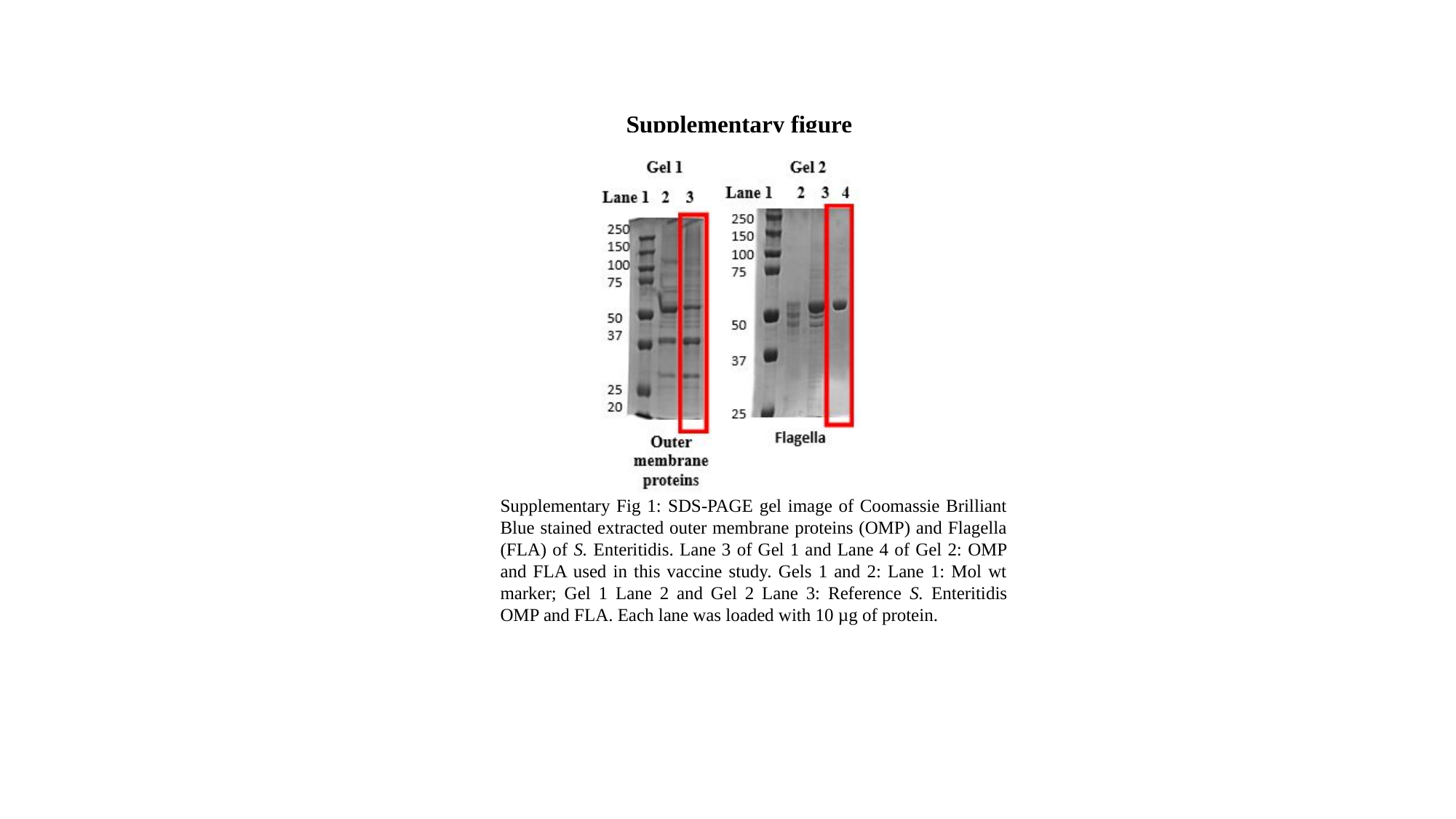

Supplementary figure
Supplementary Fig 1: SDS-PAGE gel image of Coomassie Brilliant Blue stained extracted outer membrane proteins (OMP) and Flagella (FLA) of S. Enteritidis. Lane 3 of Gel 1 and Lane 4 of Gel 2: OMP and FLA used in this vaccine study. Gels 1 and 2: Lane 1: Mol wt marker; Gel 1 Lane 2 and Gel 2 Lane 3: Reference S. Enteritidis OMP and FLA. Each lane was loaded with 10 µg of protein.
